# Chromosomal structural abnormalities and tissue-specific mosaicism: insights into false-negative noninvasive prenatal testing

**DOI:** 10.3389/fgene.2026.1845616

**Published:** 2026-07-10

**Authors:** Ning Huang, Yonghua Xu, Shujun Ding, Lu Pan, Ping Zhou, Yongyi Zou, Chuanxin Feng, Shuhui Huang, Yanqiu Liu, Bicheng Yang, Huizhen Yuan

**Affiliations:** 1 Medical Genetics Center, Jiangxi Maternal and Child Health Hospital, Nanchang, China; 2 Medical Laboratory, Jiangxi Maternal and Child Health Hospital, Nanchang, China

**Keywords:** chromosomal structural abnormality, confined placental mosaicism, copy number variations, dicentric chromosome 18, false negative, noninvasive prenatal testing, ring chromosome 21

## Abstract

**Objective:**

To investigate the causes of false-negative results in noninvasive prenatal testing (NIPT) and provide insights for technical optimization and genetic counseling in clinical practice.

**Methods:**

We retrospectively analyzed clinical and genetic data from three cases with false-negative NIPT results. In case 1, amniocentesis was performed due to fetal growth restriction (FGR) and a single umbilical artery (SUA). In case 2, a female infant was delivered at 35 weeks’ gestation for FGR and vasa previa, with postnatal manifestations including neonatal respiratory distress syndrome, low birth weight, and hexadactyly. In case 3, amniocentesis was carried out for a high-risk NIPT result indicating a 14q duplication. Genetic analyses including karyotyping, copy number variation sequencing (CNV-seq), trio whole-exome sequencing (WES-trio), and fluorescence *in situ* hybridization (FISH) were performed on amniotic fluid, placental tissue, maternal peripheral blood, and buccal mucosal samples, respectively.

**Results:**

Ring chromosome 21, dicentric chromosome 18, and multiple chromosomal rearrangements were identified in the three cases. In Case 1, amniotic fluid analysis revealed mosaic r (21) by karyotyping and a terminal deletion of 21q by CNV-seq, which was attributed to dynamic mosaicism and potential cell culture artifacts. In Case 2, karyotyping and WES detected 42% and 65% mosaic dic (18) in peripheral blood, respectively, whereas FISH on buccal mucosal cells only identified 8% mosaicism. This substantial discrepancy reflected tissue-specific mosaic distribution between buccal cells (ectoderm) and peripheral blood (mesoderm) of dic (18). In Case 3, prenatal karyotyping and CNV-seq confirmed a pathogenic 5p deletion in the fetus. Subsequent genetic testing of placental specimens revealed complex mosaic CNVs (10%–52%), whereas umbilical cord findings were consistent with amniotic fluid results. Complex chromosomal rearrangements occurring during early embryogenesis led to widespread multi-CNV mosaicism across placental and fetal tissues. Such heterogeneous genomic alterations are incompletely represented in cffDNA, resulting in concurrent false-positive and false-negative NIPT results.

**Conclusion:**

Mosaicism levels, tissue distribution variability, cellular heterogeneity, and complex chromosomal structural abnormalities are key contributors to false-negative NIPT results. Our findings highlight the importance of comprehensive clinical and genetic evaluation in managing discordant NIPT findings.

## 1 Introduction

With high sensitivity and specificity for common fetal aneuploidies, noninvasive prenatal testing (NIPT) has evolved into a first-tier prenatal screening technique by analyzing cell-free fetal DNA (cffDNA) in maternal plasma (Author Anonymous, 2016; Tian et al., 2020; Gregg et al., 2016). As the NIPT has rapidly and wildly spread, discrepancies between NIPT results and fetal genetic findings have become an inevitable challenge (Hartwig et al., 2017; Kónya et al., 2026).

The false-negative rate of NIPT for common fetal aneuploidies has been estimated to be 0.65% (Liehr, 2022). The occurrence of NIPT false-negative results stems from three core factors including fetal, maternal, and placental heterogeneous conditions. Such as inadequate fetal fraction failed to meet the sequencing threshold for effective variant identification. Maternal fators including maternal copy number variations (CNVs) and maternal malignancies which interfere with background sequencing signals. Confined placental mosaicism (CPM) represents genetic differential distribution between placenta and fetus (Lannoo et al., 2021; Galeva et al., 2025).

Chromosomal structural rearrangements combined with dynamic mosaicism are severely underestimated in conventional NIPT reports and clinical screening (Thomsen et al., 2026). NIPT cannot directly detect chromosomal structural abnormalities and only identifies these variants indirectly through chromosomal deletions or duplications. Unlike simple aneuploidies, rare structural anomalies including ring chromosomes, dicentric chromosomes, and multi-copy number variations usually present unstable embryonic mitosis, resulting in heterogeneous mosaic distribution across fetal germ layers and placental tissues, further increasing the difficulty of accurate detection and easily causing missed diagnosis and misdiagnosis in clinical practice (West and Everett, 2022). Currently, systematic clinical summaries and mechanism analyses focusing on NIPT discordance caused by chromosomal structural variants and dynamic mosaic remain limited, leading to inadequate awareness of such special cases in prenatal counseling, and standardized clinical management strategies are absent.

In this study, we systematically analyzed three NIPT discordant cases involving ring chromosome 21, dicentric chromosome 18, and complex multi-CNV mosaicism. This findings aims to supplement the theoretical basis for NIPT limitations in complex chromosomal structural anomalies, emphasize the high missed-diagnosis risk of mosaic structural variants in routine NIPT, and provide evidence-based references for technical optimization, standardized genetic counseling, and individualized clinical management of discordant NIPT cases.

## 2 Materials and methods

### 2.1 Case presentation

Case 1: A 36-year-old healthy pregnant woman was referred to the Medical Genetics Center of Jiangxi Maternal and Child Health Hospital. NIPT-plus was performed at 14 weeks of gestation, indicating a low risk with a chromosome 18 Z-score of 0.678; nuchal translucency (NT) measurements were also within the normal range. At 23 weeks of gestational age (GA), ultrasound examination revealed that the fetal biparietal diameter and head circumference were approximately 2 weeks smaller than expected for the GA, while the abdominal and limb circumferences were more than 1 week smaller than normal. Additionally, a single umbilical artery (SUA) was observed, prompting subsequent amniocentesis.

Case 2: A 27-year-old woman who conceived via *in vitro* fertilization and embryo transfer (IVF-ET) underwent prenatal screening. NIPT-plus results indicated a low risk, and NT ultrasound findings were normal. At 24 weeks’ GA, ultrasound showed fetal growth parameters approximately 1 week below the expected average, along with an abnormal structure on the lateral aspect of the right thumb and a low-lying placenta. By 31 weeks’ GA, the abdominal and limb circumferences had progressed to more than 3 weeks smaller than normal. Due to fetal growth restriction (FGR) and vasa previa, a female infant was delivered by cesarean section at 35 weeks’ GA.

Case 3: A 37-year-old woman with a history of two pregnancies was evaluated. Her first pregnancy ended in embryonic arrest at approximately 2 months of gestation. During her second pregnancy, NIPT-plus identified a high-risk result, with a duplication of chromosome 14q22.3q32.33 (approximately 48.42 Mb). Amniocentesis was subsequently performed for confirmatory diagnosis, and the results did not confirm the 14q22.3q32.33 duplication indicated by NIPT-plus, consistent with a false-positive NIPT-plus result.

Cases 1 and 2 were categorized as NIPT false-negative, defined as low-risk NIPT results whereas fetal genetic testing confirmed clinically significant chromosomal aberrations. By contrast, Case 3 showed mixed discordant outcomes: NIPT indicated a false-positive result of 14q duplication, while fetal genetic analysis confirmed a 5p14.3 deletion, thus presenting concomitant false-positive and false-negative NIPT results.

The clinical presentations are summarized in Table 1.

**TABLE 1:** Clinical presentation of three cases with false-negative NIPT results.

Case	NIPT-plus	Clinical presentation	Pregnancy outcome
1	Negative	FGR, SUA	TOP
2	Negative	FGR, vasa previa; postnatal: NRDS, LBW, hexadactyly	Delivered via cesarean section
3	High risk (14q dup)	History of embryonic arrest	TOP

FGR: Fetal growth restriction. SUA: single umbilical artery. NRDS: neonatal respiratory distress syndrome. LBW: low birth weight. TOP: termination of pregnancy.

### 2.2 Expanded non-invasive prenatal test

NIPT-plus testing was conducted with the expanded non-invasive prenatal screening kit (BGI Biotechnology, Wuhan, China) on the MGISEQ-2000 sequencing platform. The assay supports a detection resolution of ≥5 Mb for subchromosomal copy number variations. It screens trisomy 13, 18, 21, other chromosome abnormalities, and 92 clinically significant microdeletion/microduplication syndromes.

### 2.3 Karyotype analysis

3 mL of peripheral lymphocyte blood was collected from all family members. Additionally, 30 mL of amniotic fluid was obtained from Case 2 and 3, respectively. All cells were cultured and subjected to karyotyping following standard protocols. Cytogenetic reports adhered to the International System for Cytogenetic Nomenclature (ISCN 2024).

### 2.4 Copy number variation sequencing (CNV-seq)

For prenatal samples, CNV-seq was performed using DNA extracted from amniotic fluid. Given the discordance between NIPT-plus results and karyotype findings in Case 3, CNV-seq analysis was also conducted on placental and umbilical cord tissues obtained post-delivery. For placental genetic testing, multiple independent tissue fragments were collected from four distinct regions of the placenta: the placental cord insertion site, fetal surface, maternal surface, and peripheral margin of the placenta. Using a 100 kb window as the basic unit of analysis, all sequencing data were aligned to the human reference genome (GRCh37/hg19).

### 2.5 Whole-exome sequencing (WES)

Uncultured amniotic cells from Case 1 and peripheral blood samples from Case 2 were collected for DNA extraction and library preparation. Target genes of the exon region, including adjacent splice sites, were captured and enriched using the BGI V4 capture microarray. Sequencing was carried out on the MGISEQ-2000 platform for variant detection. Quality control metrics for the sequencing data met the following criteria: the average effective sequencing depth across the target region was at least 100×, and more than 95% of sites achieved a depth greater than 20×.

### 2.6 Fluorescence in situ hybridization (FISH)

According to the manufacturer’s protocol, buccal mucosal cells collected from the newborn in Case 2, subsequently hybridization at 37 °C overnight and post-hybridization washes at 45 °C to remove non-specific binding. A total of 100 cells were evaluated following hybridization with centromere-specific probes for chromosomes 18, X, and Y (An Biping, China).

### 2.7 Chimerism assessment

Additional cell counting was performed for mosaic karyotype, with each cell line counted separately to calculate the proportion of abnormal cells. For CNV-seq and WES, skewed chromosomal copy numbers were used to infer the presence of genomic mosaicism.

## 3 Results

### 3.1 Genetic anomalies

**FIGURE 1:**
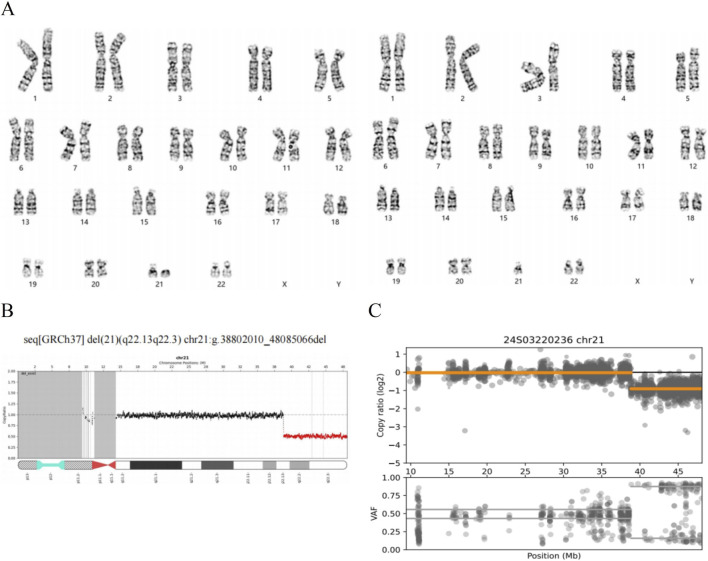
The fetal genetic results in Case 1. Fetal karyotype analysis revealed a mosaic ring chromosome 21, CNV-seq and WES identified a 9.28 Mb deletion at 21q22.13-q22.3. The fetal sex chromosomes are not disclosed. 46,U replaces 46, XX or 46, XY. **(A)** 46, U, r(21) (p13q22) [53]/45, U,-21[7] confirmed in amniocytes karyotyping. **(B)** del(21) (q22.13q22.3).seq[GRCh37/hg19](38802010_48085066)×1 detected in amniocytes CNV-seq. **(C)** del(21) (q22.13q22.3).seq [GRCh37/hg19] (38828579-48119395) × 1 detected in amniocytes WES.

In Case 1, amniocentesis was performed due to FGR and SUA, as described in Section 2.1. Fetal karyotype analysis revealed a ring chromosome 21: 46,U,r (21) (p13q22)[53]/45,U,-21 (Lannoo et al., 2021). CNV-seq identified a 9.28 Mb deletion at 21q22.13–q22.3 (Figure 1), and WES-trio confirmed this deletion was a *de novo* mutation, with no corresponding abnormalities detected in the parents.

**FIGURE 2:**
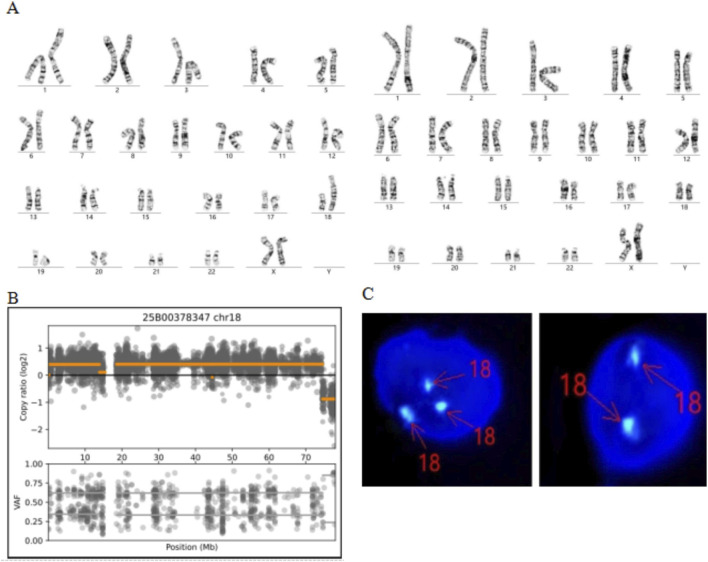
The genetic results of the newborn in Case 2. Karyotyping and WES identified a dicentric chromosome 18 with mosaicism 42% and 65%, respectively. While FISH analysis on buccal mucosal cells detected only 8% trisomy 18. **(A)** 46, XX, psu idic(18) (q23) [21]/46, XX [29] in peripheral blood for karyotyping **(B)** dup (18p11.32q23).seq [GRCh37/hg19] (192841-74534063) × 2.55, del (18q23).seq [GRCh37/ hg19] (74536313-78016748) × 1 in peripheral blood for WES **(C)** CSP18 × 3[8]/CSP18 × 2 [92] buccal mucosa FISH by centromere-specific probes for chromosomes 18.

In case 2, a female infant was delivered via cesarean section at 35 weeks of gestation due to FGR and vasa previa. Postnatal manifestations included neonatal respiratory distress syndrome, low birth weight, and hexadactyly. Cytogenetic analysis of peripheral blood lymphocytes identified a dicentric chromosome 18: 46,XX,psu idic (18) (q23) (Ye et al., 2024)/46,XX (Eggenhuizen et al., 2024), with 42% mosaicism for idic (18). WES revealed a deletion at 18q23, along with a mosaic duplication of 18p11.32–q23 (estimated copy number: 2.55; mosaicism level: 65%). In contrast, FISH analysis on buccal mucosal cells detected only 8% mosaic cells, indicating a significant tissue-specific discrepancy in mosaicism level (Figure 2).

**FIGURE 3:**
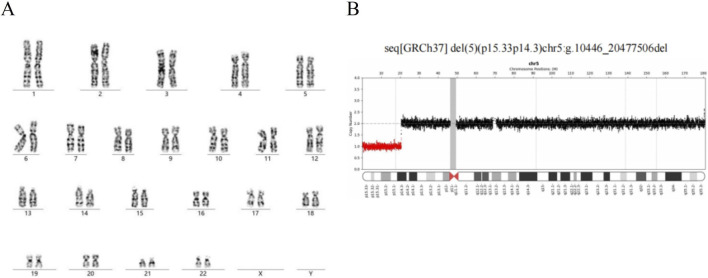
The fetal genetic results in Case 3. The fetal sex chromosomes are not disclosed. 46,U replaces 46,XX or 46,XY. **(A)** 46, U, del (5) (p14.3) in amniotic fluid for karyotyping. **(B)** del (5) (p15.33p14.3).seq [GRCh37/hg19] (10446-20477506) × 1 20.47 Mb in amniotic fluid for CNV-seq.

In Case 3, amniocentesis was prompted by a high-risk NIPT-plus result indicating a 14q duplication. Subsequent karyotyping and CNV-seq of amniocytes identified a pathogenic 5p14.3 deletion that had been entirely missed by NIPT-plus, representing a false-negative and false-positive result. Following pregnancy termination, placental CNV-seq uncovered multiple mosaic CNVs (10%–52% mosaic ratio), including dup (14q), dup (1q), del (5p), dup (17q), dup (16q) and dup (10q). Umbilical cord blood detected del (5) (p14.3), consistent with amniotic fluid results, verifying the fetal 5p deletion was genuine, whereas the 14q duplication detected only in mosaic placenta failed to manifest in fetal somatic tissue, and the fetal 5p deletion not reflected in cffDNA (Figure 3).

The above genetic results are summarized in Table 2. Notably, no genetic abnormalities were detected in either parent of the three cases.

**TABLE 2:** Cytogenetic and molecular genetic findings in the three cases.

Case	Sample type	Karyotype	CNV-seq	WES-trio	FISH
1	Amniotic fluid	46,U,r (21) (p13q22)[53]/45,U,–21 [7]	del (21) (q22.13q22.3) ∼9.28 Mb	Del (21) (q22.13q22.3) ∼9.29 Mb	–
2	Peripheral blood	46,XX,psu idic (18) (q23) [21]/46,XX[29]	–	Dup (18p11.32q23)×2.55, del (18q23)×1	–
	Buccal mucosa	–	–	–	CSP18 × 3 [8]/CSP18 × 2 [92]
3	Amniotic fluid	46,U,del (5) (p14.3)	Del (5) (p15.33p14.3) 20.47 Mb	–	–
	Placental tissue	–	Multiple mosaic CNVs (mosaicism 10%–52%): Dup (14q22.3q32.33) ∼51.54 Mb Dup (1q41q43) ∼19.08 Mb Del (5p15.33p14.3) 20.45 Mb Dup (17q22q25.3) ∼24.58 Mb Dup (16q22.1q24.2) ∼18.92 Mb Dup (10q22.1q26.2) ∼56.90 Mb	–	–
	Umbilical cord	–	Del (5) (p15.33p14.3) 20.46 Mb	​	​

Represent the corresponding technique was not applied.

### 3.2 Clinical follow-up

Following the identification of pathogenic genetic anomalies, the pregnancies in Cases 1 and 3 were terminated at 26 and 22 weeks of gestation, respectively. For Case 1, the patient subsequently conceived naturally. Prenatal diagnostic testing will be planned during her second trimester. For Case 3, given her history of two adverse pregnancy outcomes, preimplantation genetic testing (PGT) was performed and amniocentesis confirmed the absence of pathogenic CNVs in the fetus, and a male infant was delivered by cesarean section at 39 weeks of gestation, with a birth weight of 3.2 kg and an Apgar score of 9.

For Case 2, the child (now 7 months old, corrected gestational age approximately 6 months), underwent surgical correction for hexadactyly postnatally. Postnatal evaluations, including cardiac functions with Doppler echocardiography and cranial magnetic resonance imaging (MRI), revealed no significant structural abnormalities. At present, the child exhibits normal neurological responses, with no dysmorphic facial features or evident developmental anomalies.

## 4 Discussion

Previous studies investigating non-maternal contributors to false-negative NIPT results have predominantly centered on confined placental mosaicism (CPM), fetal mosaicism, and the relationship between detection sensitivity and copy number variants (CNVs) fragment size (Liang et al., 2019; Tian et al., 2023). In contrast, false negatives attributable to chromosomal structural abnormalities have limited attention. In this study, we present three cases of false-negative NIPT associated with rare chromosomal structural abnormalities: ring chromosome 21, dicentric chromosome 18, and a 5p deletion with confined placental mosaicism (CPM) involving multiple CNVs.

Ring chromosomes are rare chromosome structural anomalies, with a reported neonatal incidence of 1 in 50,000 according to the International Consortium for Human Ring Chromosomes (Sobol et al., 2024; Pristyazhnyuk and Menzorov, 2018). Bone K reported a false-negative NIPT case involving r (21), and amniocytes karyotyping revealed only a single r (21) cell line. Array comparative genomic hybridization (aCGH) and quantitative fluorescence polymerase chain reaction (QF-PCR) detected both a terminal 21q deletion and mosaic duplication of the remaining chromosome 21 segments (Bone et al., 2019). Notably, however, in Case 1 Karyotyping identified r (21) and monosomy 21, whereas CNV-seq and WES only detected a 21q deletion. These discordant results can be explained that ring chromosome is inherently unstable and often generates dynamic mosaicism through mitotic missegregation (Ambulk et al., 2023; Kushwaha et al., 2024; Petter et al., 2019). Theoretically, mosaicism involving ring chromosomes is expected to involve multiple cell lines. However, these unstable cell lines, such as double r (21) and dic r (21), are prone to loss during cell culture (Li et al., 2022). As a result, CNV-seq and WES only detected a distal long-arm deletion of chromosome 21 (approximately 9.28 Mb), which was not detected by NIPT. Our findings highlight the limited ability of NIPT to detect submicroscopic terminal deletions. The unstable nature of dynamic mosaic ring chromosome 21 ultimately led to discordant results across karyotyping, CNV-seq, WES and NIPT.

Meta‐analysis for trisomy 18 reported 2.07% rate of false-negative results in a 647,442 NIPT cohort (Liehr, 2022). First-trimester ultrasound (ideally at 11–13^+6^ weeks’ gestation) may detect characteristic features of trisomy 18, most commonly increased nuchal translucency, as well as cardiac and extracardiac anomalies (Ye et al., 2024; Chen, 2024). While in Case 2, we presented a newborn with mild dysmorphic features (hexadactyly) and a rare mosaic partial trisomy 18 variant: 1 cell line harbored a dicentric chromosome 18 with a 3.48 Mb terminal deletion of 18q23 and a 74.34 Mb duplication of 18p11.32q23. Sheehan E reported four false-negative NIPT cases of full trisomy 18 with multiple fetal ultrasonographic anomalies (Sheehan et al., 2023). The proportion and tissue distribution of trisomy 18 cells are closely correlated with the clinical phenotypic severity. Facing a newborn with mosaic partial trisomy 18, comprehensive assessment of mosaicism distribution and levels across multiple tissues is critical to guide clinical management and identify the cause of false-negative NIPT results. In our case, FISH with chromosome 18 centromeric probes on buccal mucosal cells detected three signals in 8% cells, in contrast to karyotyping 42% and WES 65%. The discrepancy primarily stems from differences in detection principles and resolution: Karyotyping involves single-cell analysis and can detect the coexistence of normal and abnormal cells; WES, as a bulk sequencing method on mixed DNA, reflects only the overall average copy number, where signals from a small number of normal cells are masked. Additionally, the unstable segregation of the dicentric chromosome 18 further contributes to the discrepancy. These findings highlight the additional value of non-invasive FISH analysis on buccal mucosal cells for evaluating mosaic aneuploidies, particularly in infants: this approach can help clarify the biological causes underlying false-negative NIPT results and the heterogeneous distribution of different germ layers in newborn (Graff et al., 2020; Ossama et al., 2023).

Previous studies have established that dynamic genomic mosaicism commonly occurs during early human embryonic development—forming the foundational biological mechanism underlying confined placental mosaicism (CPM) (Zhai et al., 2024). Both fetal mosaicism and CPM are major contributors to false-negative NIPT results. In contrast, CPM in conjunction with maternal chromosomal aberrations is a leading cause of NIPT false positives (). By contrast, Case 3 represents a rare composite case with concurrent NIPT false-positive and false-negative results. Multiple copy-number abnormalities were identified in placental specimens, indicating that complex chromosomal rearrangements arising in early embryogenesis drive the coexistence of diverse mosaic CNVs. Furthermore, complex multi-chromosomal structural rearrangements can result in divergent cytogenetic compositions across different tissues (the placenta, umbilical cord, and amniotic fluid). NIPT detects placenta-derived cell-free DNA and is not directly governed by the true fetal genotype. In Case 3, due to placental multi-clone mosaicism, only the 14q duplication clone was present at sufficiently high abundance in the trophoblast to release enough abnormal DNA into the maternal circulation, leading to a false-positive result. The abnormal clone corresponding to the fetal 5p deletion was of insufficient abundance in the placenta, so its abnormal signal was diluted and missed. The other various duplication clones in the placenta either were not in the trophoblast or had too low a proportion to reach the detection threshold of NIPT. Consequently, only the 14q duplication tested positive. Placental-fetal genomic discordance, characterized by high-level placental mosaicism for multiple CNVs not fully reflected in cffDNA, constitutes a key mechanism contributing to false-positive and false-negative NIPT results (Eggenhuizen et al., 2024).

Unlike common aneuploidies, rare chromosomal structural aberrations—including ring chromosomes, dicentric chromosomes, and complex chromosomal rearrangements—induce mitotic instability and dynamic genomic mosaicism (Wang et al., 2025), leading to pronounced inter-tissue genomic heterogeneity among placental, amniotic, and fetal germ layers. These tissue-specific genomic disparities frequently yield discordant results across different genetic testing platforms and necessitate integrated cytogenetic and molecular analyses for definitive prenatal diagnosis (Zhang et al., 2026).

Our study provides evidence that complex structural lesions, variable mosaic fractions, and spatially restricted genomic heterogeneity impair NIPT accuracy and constitute a key biological basis for false-negative outcomes. Clinicians should prioritize comprehensive pre- and post-test genetic counseling, leverage prenatal ultrasonography as an early phenotypic sentinel, and adopt a multimodal diagnostic strategy—integrating ultrasound with complementary cytogenomic assays—to detect occult fetal anomalies, elucidate mechanisms underlying test discordance, and enable individualized prenatal and postnatal management (Gadsbøll et al., 2024; Stefanovic, 2019; Andrietti et al., 2026).

## Data Availability

The original contributions presented in the study are publicly available. This data can be found here: https://doi.org/10.6084/m9.figshare.32916758.
